# 

**Published:** 1886-05

**Authors:** 


CONTENTS

VOL. 33 No. 5

| | PAGE. |
|---|---|
| Clairvoyance | 129 |
| Mental Cure | 132 |
| Yellow Fever | 133 |
| Praying and Dieting | 134 |
| Ottorrhœ—Its Rational Treatment | 135 |
| The Principle of Heavy Clothing | 139 |
| Cremation in France | 141 |
| Oxygen in Therapeutics | 142 |
| The Topical use of Earths | 144 |
| Importance of Dietetics | 146 |
| Scarlet Fever | 147 |
| Death of one of Pasteur's Patients | 148 |
| Malaria | 149 |
| Tenabine | 150 |
| Training School for the Insane | 151 |
| Natural Healing | 152 |
| Treatment of Sprains | 153 |
| Doctors Disagree | 154 |
| Bleeding at the Nose | 155 |
| A Healthy Diet | 155 |
| Miscellaneous Items | 156 |
| Home Department | 157 |

INDEX TO ADVERTISEMENTS OF HALF A PAGE AND OVER.

| | |
|---|---|
| Teller's Hypo Phosphate | I |
| Lactopeptine | II |
| Castoria | II |
| Hosford's Acid Phosphates | III |
| Caulocorea | III |
| Medicated Supposatories | IV |
| Lacteal Fluids | VII |
| Perforated Paper | inside of cover |
| Compound Oxygen | last page of cover |

